# Detection of soft tissue foreign bodies by nurse practitioner-performed ultrasound

**DOI:** 10.1186/2036-7902-6-2

**Published:** 2014-01-29

**Authors:** Paul Atkinson, Rajeev Madan, Richard Kendall, Jacqueline Fraser, David Lewis

**Affiliations:** 1 Emergency Medicine, Saint John Regional Hospital, Saint John, NB E2L 4L4, Canada; 2 Emergency Medicine, Dalhousie University, Halifax, NS B3H 4R2, Canada; 3 Emergency Medicine, Memorial University, St John’s, NF A1B 3X9, Canada; 4 Emergency Medicine, Cambridge University Teaching Hospitals, Cambridge, Cambridgeshire CB2 0QQ, UK

**Keywords:** Nurse practitioners, Point-of-care ultrasound, Wound care, Foreign bodies, Diagnosis

## Abstract

**Background:**

This study aimed to evaluate the accuracy of emergency nurse practitioner (NP)-performed point-of-care ultrasound (POCUS) for the detection of soft tissue foreign bodies (FBs).

**Methods:**

Following a 2-h training session, ten NPs were assessed on their ability to detect various FBs in an experimental model. FBs (wood, metal and plastic) were inserted randomly into eight experimental models (uncooked chicken thighs) by an independent observer. Control experimental models had no FB inserted, but all had a 1-cm incision made on their surface. NPs, blinded to the type of model, were then assessed on their ability to detect the FBs by ultrasound examination using high-frequency linear transducers (Toshiba Nemio). Models were also scanned by two experienced emergency physicians (EPs) as a further control.

**Results:**

Overall, NP-performed POCUS detected 47 of the 60 foreign bodies with a sensitivity, specificity, positive predictive value and negative predictive value of 78.3%, 50%, 82% and 43%, respectively, compared with 83.3%, 75%, 90.9% and 60% for EPs. Sensitivity for detecting specific types of FB was 95%, 85% and 50% for wood, metal and plastic, respectively, for NP-performed POCUS, compared with 100%, 100% and 50% in the EP group.

**Conclusions:**

NPs with no previous ultrasound experience can detect soft tissue FBs with accuracy comparable to that of EPs in an experimental model. Test sensitivity was high for wood and metal foreign bodies. Specificity was generally low.

## Background

Point-of-care ultrasound (POCUS) is widely used by emergency physicians and radiologists for the detection of soft tissue foreign bodies (FBs) [1].

Previous studies have demonstrated that POCUS has reasonable accuracy for detection of soft tissue FBs, with a range of sensitivities of 69% to 88% and specificities of 59% to 90% for soft tissue foreign bodies [2-4], and that there is a similar accuracy between different types of sonographers, with sensitivities of 83%, 85% and 74% and specificities of 83%, 85% and 87% for radiologists, ultrasound technicians and emergency physicians, respectively [2], and between emergency physicians with a sensitivity of 97% and a specificity of 70% and trainees with a sensitivity of 86% and a specificity of 83% [3].

The accuracy of POCUS to detect FBs differs for various substances with one study showing sensitivities for wood of 93% and plastic of 73% [4].

Nurses and nurse practitioners are increasingly using POCUS for focused assessment and procedures in the emergency department setting. There is growing evidence that with appropriate training, nursing staff can use POCUS safely [5,6].

Much of the literature supporting nurse- and nurse practitioner-performed POCUS is in the setting of traditional core applications of emergency POCUS such as focused assessment by sonography in trauma (FAST) scanning and ultrasound-guided vascular access [6]. Many nurses and nurse practitioners practice more independently in the minor injury setting in urgent care centres and emergency department fast tracks or rapid assessment zones, where they perform initial wound care. Undetected soft tissue FBs can cause delayed wound healing and infection [7]. This study aimed to evaluate the accuracy of emergency nurse practitioner (NP)-performed POCUS for the detection of soft tissue foreign bodies.

## Methods

### Study design

The study design was a prospective blinded experimental diagnostic test study. The study was reviewed by the chair of the local research ethics committee, and need for formal approval was waived.

### Study setting and population

The training course and experimental testing were performed at a major tertiary care teaching hospital. Faculty and supervisors were fully accredited by the College of Emergency Medicine in Emergency Point of Care Ultrasound, holding the Certificate in Focused Emergency Ultrasound (CFEU). The nurse practitioners were also staff at the same facility, having achieved full nurse practitioner status and were all actively clinically in the Emergency Department with an annual census of over 90,000 visits per year.

### Study protocol

Ten nurse practitioners each underwent a 2-h hands-on training session by a certified POCUS instructor. This training session consisted of pre-course reading material, a short didactic component (covering the basics of ultrasound physics, instrumentation and the use of ultrasound for detection of soft tissue foreign bodies), and supervised bedside teaching using volunteer models and experimental models (described below). Both the normal ultrasound anatomy of the experimental model and the ultrasound appearance of the various foreign bodies were demonstrated.

**Figure 1:**
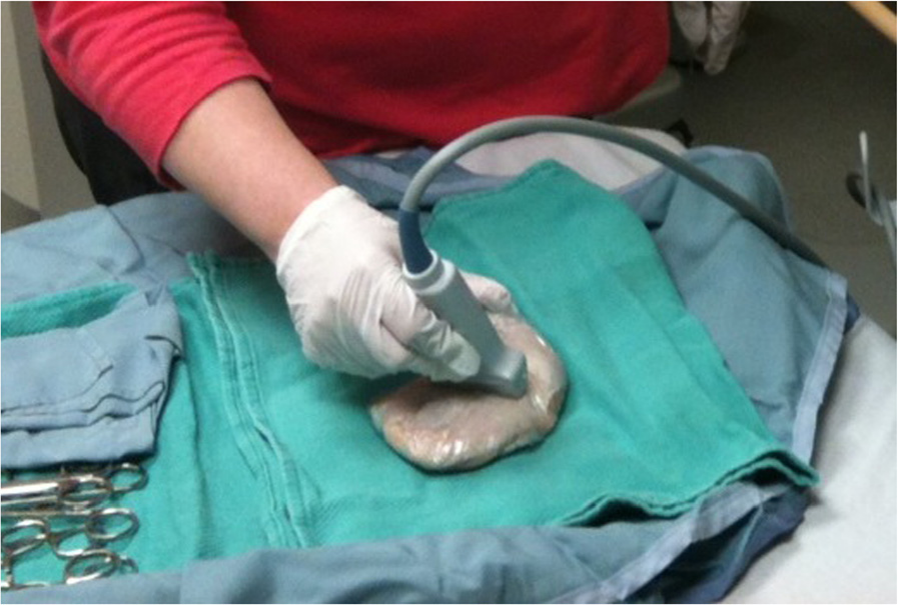
Experimental model consisting of plastic-wrapped chicken thighs containing FBs or none, with a 1-cm incision.

In a separate session, following initial training, each NP was individually assessed on their ability to detect various FBs which had been pre-inserted into in an experimental model. The experimental models consisted of wrapped (cling wrap) uncooked chicken thighs each containing either a single metal, glass or plastic FB or no FB (Figure 1). All models were numbered for identification.

**Figure 2:**
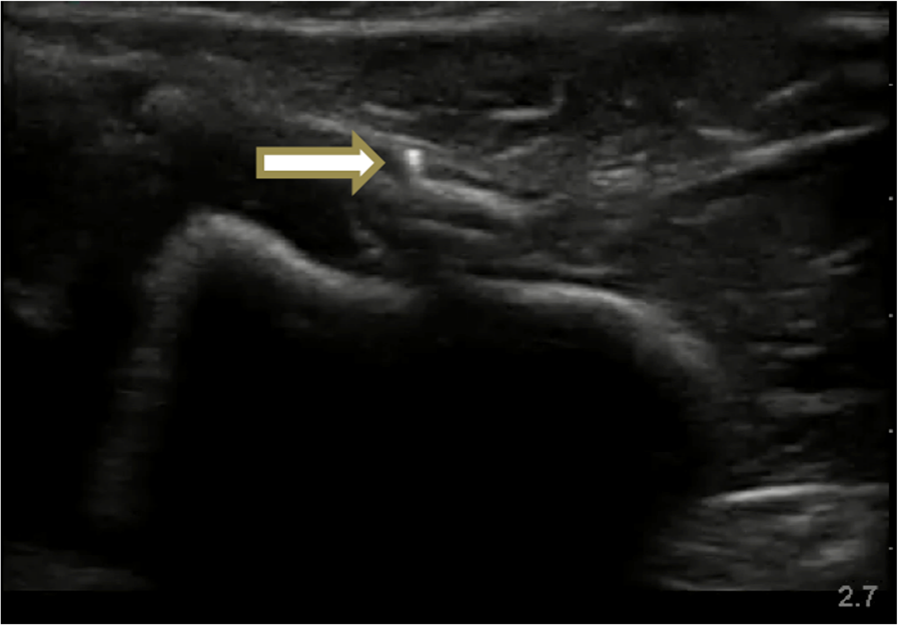
Metal foreign body (arrow) as seen by ultrasound.

Wood, metal and plastic FBs were inserted randomly by an independent observer (also a trained POCUS instructor) into the muscle layer of eight experimental models at a depth of 10 to 15 mm. FBs measured 15 to 20 mm in length and 1 to 3 mm in diameter. Control experimental models had no FB inserted. The independent observer recorded which materials were present in each model. All models had a 1-cm incision made on their surface. NPs, blinded to the type of model, were then assessed on their ability to detect the FBs by ultrasound examination (Figure 2) using high-frequency linear transducers (Toshiba Nemio, Toshiba Medical Systems, Tokyo, Japan). For each model, they were asked to record whether an FB was present or absent.

All experimental models were also scanned by two experienced emergency physicians (EPs) as a further quality control measure. These EPs were blinded to the model contents in the same fashion as the NPs. Assessment of accuracy was performed by a third unblinded EP.

Statistical analyses of test characteristics were performed using MedCalc for Windows, version 12.5 (MedCalc Software, Ostend, Belgium).

## Results

Nurse practitioners performed a total of 80 scans, and emergency physicians performed 16 scans (see Table 1). NP-performed POCUS had a sensitivity of 0.783 (95% confidence interval (CI) 0.664 to 0.869) with a specificity of 0.50 (95% CI 0.299 to 0.701) for detection of soft tissue foreign bodies in the experimental models. EP-performed POCUS had a sensitivity of 0.833 (95% CI 0.552 to 0.953) with a specificity of 0.75 (95% CI 0.301 to 0.954). Further descriptive diagnostic test statistics for NP- and EP-performed POCUS are shown in Table 2. The sensitivity of both NP- and EP-performed POCUS for different types of foreign bodies was also calculated. NP-performed POCUS had sensitivities of 95%, 85% and 50% for wood, metal, and plastic foreign bodies, respectively. EP-performed POCUS had sensitivities of 100%, 100% and 50% for wood, metal, and plastic foreign bodies, respectively.

**Table 1:** Summary of diagnostic test findings for NP- and EP-performed ultrasound for detection of soft tissue FBs

	**Disease positive**	**Disease negative**	**Totals**
NP			
Test positive	47	10	57
Test negative	13	10	23
Totals	60	20	80
EP			
Test positive	10	1	11
Test negative	2	3	5
Totals	12	4	16

**Table 2:** Descriptive statistics for NP- and EP-performed ultrasound as a test for detection of soft tissue FBs

	**NP**		**EP**	
	**Estimate**	**95% CI**	**Estimate**	**95% CI**
Sensitivity	0.783	0.664 to 0.869	0.833	0.552 to 0.953
Specificity	0.5	0.299 to 0.701	0.75	0.301 to 0.954
PPV	0.825	0.706 to 0.902	0.909	0.623 to 0.984
NPV	0.435	0.256 to 0.632	0.6	0.231 to 0.882
LR+	1.567	0.991 to 2.477	3.333	0.599 to 18.543
LR-	0.433	0.226 to 0.831	0.222	0.056 to 0.889

## Discussion

This experimental study describes the diagnostic accuracy of nurse practitioner-performed POCUS for the detection of FBs in an experimental model. Using a small group of experienced emergency physicians as an experimental validity control measure, we have demonstrated that following a relatively short and focused period of training, nurse practitioners demonstrated an ability to perform POCUS screening for imbedded soft tissue foreign bodies with reasonable levels of sensitivity. In this experimental model, the sensitivity (0.783; 95% CI 0.664 to 0.869) and specificity (0.50; 95% CI 0.299 to 0.701) of NP-performed POCUS compare favourably with historical controls (sensitivity range of 0.69 to 0.88 and specificity range of 0.59 to 0.90). It is also apparent that some types of FB (wood and metal) are more easily detected than others (plastic).

### Limitations

It is difficult to conclude that the study shows clinical competence, as the findings were limited to experimental chicken models with artificially inserted foreign bodies. This however is the technique used to train emergency physicians in several accredited courses [8,9].

Other limitations include the time limitation of both the training and testing. The authors’ personal experience indicates that the time taken to identify FBs on PoCUS is not uniform and may require prolonged scanning time. Also, this study has low participant numbers, especially on the emergency physician arm, leading to an unbalanced ‘control’. This control arm was used as a quality control measure, rather than a true comparative control arm.

## Conclusions

With relatively short focused training, NPs with no previous ultrasound experience can detect soft tissue FBs with accuracy comparable to that of historical controls and EPs in an experimental model.

Test sensitivity was high for wood and metal foreign bodies. Specificity was generally low.

NP-performed POCUS for detection of foreign bodies needs to be evaluated further in a clinical setting before recommending this as an extension of practice.

## Competing interests

All authors have received honoraria for teaching on Emergency Critical Care Ultrasound (ECCUCourse.com) educational ultrasound courses in the UK and Canada.

## Authors’ contributions

PA, RM and RK designed the protocol, implemented the experimental model teaching and testing, performed the literature review, drafted the manuscript and reviewed the manuscript. JF and DL reviewed the protocol, contributed to the literature review, drafted the manuscript and reviewed the manuscript. All authors read and approved the final manuscript.
